# Variation in the Reported Management of Canine Prolapsed Nictitans Gland and Feline Herpetic Keratitis

**DOI:** 10.3390/vetsci5020054

**Published:** 2018-06-01

**Authors:** Constance N. White, Gareth Jones, Sarah Baker, Rachel S. Dean, Marnie L. Brennan

**Affiliations:** 1 Fremont Veterinary Clinic, Portland, OR 97213, USA; doctornev2000@yahoo.com; 2 Park Veterinary Group, 162 Dominion Rd, Glenfield LE3 8JA, UK; Gareth.Jones@parkvetgroup.com; 3 3f2 24 Bread St, Edinburgh EH3 9AF, UK; s-j1390@hotmail.co.uk; 4 Centre for Evidence-Based Veterinary Medicine, School of Veterinary Medicine and Science, The University of Nottingham, Sutton Bonington Campus, Loughborough LE12 5RD, UK; Rachel.Dean@nottingham.ac.uk

**Keywords:** cherry eye, nictitans prolapse, third eyelid gland, feline herpes keratitis, treatment variation, clinical vignette

## Abstract

Treatment variation in medicine may be driven by evidence gaps, clinician factors, and patient preferences. Although well-documented in human medicine, variation in clinical management is relatively unexplored in veterinary practice. Clinical vignette questionnaires were administered to a cross section of general practitioners (GPs) and veterinarians with postgraduate training in ophthalmology (PGs) to survey recommended management of canine prolapsed nictitans gland (“cherry eye”, PNG) and feline herpesvirus (FHV-1) keratitis. The majority of veterinarians (96.2%) suggested surgical replacement of cherry eye, with a pocketing technique being the most frequently nominated procedure. GPs were more likely to suggest gland excision in the event of surgical failure, while PGs more frequently nominated techniques incorporating a periosteal anchor for salvage repair. Most respondents managed FHV-1 keratitis with topical antibiotics (76.4%), with a minority suggesting topical antivirals (32.2%). GPs favoured topical acyclovir whilst PGs more frequently recommended topical trifluorothymidine. A significantly larger proportion of PGs nominated systemic famciclovir and lysine supplement for FHV-1 keratitis. This survey revealed moderate treatment variation for these conditions, both between and within practitioner groups. Additional research is needed to assess the reasons for this variation, particularly for conditions in which high quality evidence is scant.

## 1. Introduction

Prolapsed nictitans gland (“cherry eye”, PNG) of dogs is a condition commonly encountered by veterinarians, particularly in predisposed breeds [1,2]. Multiple surgical procedures for correction have been described in the veterinary literature which require varying surgical proficiency and equipment [3] (pp. 963–964). Gland excision has been discouraged due to recognition of the gland’s contribution to tear production and a study showing higher risk of keratoconjunctivitis sicca (KCS) in dogs after excision [3] (p. 963) [4] (p. 80) [5] (p. 163) [6] (p. 206) [7] . Apart from this fiat, we are unaware of any directive guidance for management of cherry eye, with surgical technique currently considered a matter of personal preference [3] (p. 964). Similarly, feline herpesvirus (FHV-1) may result in morbid and relapsing corneal and conjunctival disease in cats. In contrast to cherry eye, definitive diagnosis of FHV-1 keratitis is challenging even with molecular testing [8,9]. No veterinary drugs are labelled for herpetic keratitis and there is little clinical research to guide treatment decisions in naturally occurring disease [8,9]. 

In human medicine, greater treatment variation may occur for conditions which lack high level evidence or guidelines [10]. Vignette-based questionnaires have been used to assess treatment patterns and variation in human clinical practice, as well to identify areas of clinical uncertainty [11,12,13]. Use of open, rather than closed, questions in vignettes may provide better insight into actual current practice [14]. We are unaware of any published literature documenting treatment patterns of these two ocular disorders in first opinion or ophthalmology practice. Ophthalmology practice patients may differ from primary care in a number of ways: cases may vary in severity, as well as available owner resources and practice capabilities. Heterogeneous management strategies may highlight resource and evidence gaps encountered by veterinarians in the treatment of these conditions and identify areas of priority for research in veterinary ophthalmology. The aim of this study was to survey veterinarians about their management of PNG in dogs and herpetic keratitis in cats. Additionally, we sought to explore variation in treatment amongst all veterinarians and between veterinarians in general practice (GPs) and those with additional ophthalmology training (PGs), with reference to published evidence regarding the treatment of these conditions.

## 2. Materials and Methods

### 2.1. Sampling and Data Collection

The target population was all members of the veterinary profession in the UK treating small animal patients. The sampling frames were a convenience sample of veterinarians on a mailing list for the Centre for Evidence-based Veterinary Medicine (CEVM) and attendees at the British Association of Veterinary Ophthalmologists (BrAVO) Winter Conference (2012).

Questionnaires (Supplementary Materials) were constructed consisting of open and closed-end questions across five sections. These sections covered the diagnostic tools used for ophthalmological cases, the sources of information accessed by vets, and factors considered in clinical decision-making, as well as questions relating to respondent demographics. The additional two sections presented two clinical vignettes—the first based on a Lhasa Apso with a PNG and the second a domestic shorthaired cat with dendritic herpetic keratitis. After each vignette, veterinarians were asked what treatments, additional investigations, long term management and recheck advice they would give for each case. Design of the vignettes was based on “textbook” cases to minimize diagnostic confusion while the associated questions were derived from a similar survey undertaken [15]. Questionnaires were pre-tested by eight individuals not engaged in the veterinary profession, and piloted by ten veterinarians engaged in academic and private practice. 

The online questionnaire was constructed and administered through cloud-based survey software (Survey Monkey Inc., San Mateo, CA, USA) using an email list of interested respondents collected from a previous survey conducted by the Centre for Evidence-based Veterinary Medicine [16]. Online respondents were encouraged to fill out the questionnaire by being entered into a prize draw for a £80 gift voucher in exchange for their participation; respondents were anonymized prior to analysis. The online survey was initiated in October 2012 and closed in November 2012. A first reminder was sent 10 days after the initial email, followed by a final reminder two days before survey close. Paper questionnaires with identical vignette, treatment, diagnostic, and ancillary management questions were distributed to the attendees of the British Association of Veterinary Ophthalmologists Winter conference and were collected back by two authors (MB, SB) at the end of the day (3 November 2012). 

### 2.2. Data Management and Analysis

Returned online surveys were downloaded to a spreadsheet (Microsoft Excel) whilst paper survey responses were manually entered into the same spreadsheet. The data from every 10th questionnaire manually entered was checked for any transcription errors. Minimal errors were encountered. Data relating to proposed treatments and diagnostic investigations were extracted from open ended responses by one coder (CNW) and categorically classified as to generic drug name or category, surgical or procedural interventions, diagnostic tests, and other patient assessments. Data related to long term recommendations were extracted by one coder (CNW) and classified as categorical data regarding prognosis, salvage treatment options, chronicity, and owner communications. Level of training in veterinary ophthalmology was assessed by questionnaire and de-anonymized email addresses after data extraction and coding. Data relating to the sources of information accessed by vets and factors considered in clinical decision making are not reported here but will appear in an additional manuscript.

Statistical analysis was performed with a commercially available statistical package (Stata IC13). Continuous data (age, years since graduation, recheck intervals) were assessed for normality by the Shapiro–Wilk normality test and were subsequently analyzed by using Mann–Whitney U tests. Chi-square tests were used to compare categorical data between groups except when expected cell counts were ≤5, where the more conservative Fisher’s exact test was used. Correction for multiple comparisons was done using the Dunn–Bonferroni method [17]. Not all respondents answered all questions; proportions are calculated using the total numbers of respondents completing each question unless otherwise indicated. Statistical significance was set at the 0.05 level. Significant p values are reported in text when not included in tables.

Ethical approval for the study was received from the ethics committee at the School of Veterinary Medicine and Science at the University of Nottingham.

## 3. Results

### 3.1. Response Rate

Of 1412 successful email invitations, 269 (18.9%) online surveys were submitted. Of 101 questionnaires distributed to British Veterinary Ophthalmologist Association (BrAVO) conference attendees, 57 (56.4%) completed questionnaires were collected. Of the total number of eligible responses received from the online cohort, 259 were engaged in general practice (from here on known as ”GPs”) while 10 had or were training for a postgraduate certificate in veterinary ophthalmology. Those 10 were combined with 50 BrAVO attendees to form a cohort with postgraduate training in veterinary ophthalmology (from here on known as postgraduate training group, “PGs”). Remaining BrAVO attendees who had not enrolled in or completed a postgraduate ophthalmology training course (n = 7) were combined with the GP online cohort. Not all 326 respondents answered all questions within the questionnaire (Table 1). 

### 3.2. Respondent Characteristics

Fifty eight percent of respondents reporting gender were female. Overall median age of respondents was 40 years (Table 2). Median age and gender distribution of PGs as compared to GPs was not significantly different, although female PGs were marginally more likely to be older than female GPs. When de-anonymized by email address subsequent to analysis, a majority of PG respondents were engaged partly (15%) or exclusively (75%) in referral practice. Credentials were verified for the 60 PG respondents: RCVS Specialist (DVOphthal, DipECVO, FANZCVS, n = 9), Certificate of Veterinary Ophthalmology (CertVOphthal, n = 38), Post Graduate Certificate in Ophthalmology (GPCert or PgCert, n = 8), GPCert/PgCert Ophthalmology candidates (n = 2), unnamed certificate in ophthalmology (n = 1), post-graduate research or training in veterinary ophthalmology (n = 2).

### 3.3. Prolapsed Nictitans Gland

The majority of both groups recommended surgical replacement of a prolapsed gland (Table 3). PGs were more likely to specify use of a pocket procedure while GPs were less likely to specify type of surgical procedure. Periosteal anchoring was mentioned by a small number of respondents as an alternative to pocketing but was rarely recommended as the sole initial technique. A small but significantly larger proportion of GPs considered gland excision as sole or alternative therapy for the initial episode of prolapse. A greater proportion of GPs recommended a trial of medical therapy prior to surgical intervention, although the difference did not reach statistical significance after correction for multiple comparisons. The most common therapies recommended for medical therapy were topical steroids (n = 32), manual reduction (n = 24), topical antibiotic (n = 16), and topical lubricant (n = 9).

**Figure 1:**
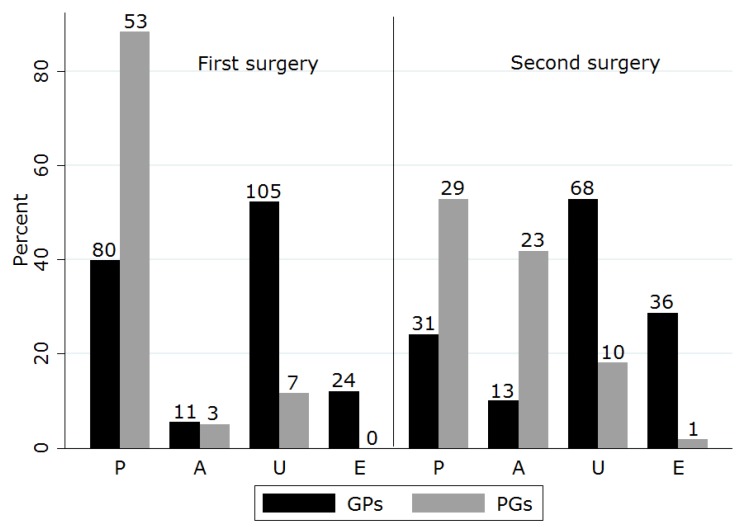
Surgical options nominated for treatment of prolapsed nictitans gland on first occurrence and for recurrence (P, pocket suggestion; A, anchoring suggestion; E, excision; U, unspecified replacement surgery suggestion).

Revision surgery suggestions for surgical failure were more varied and proportions for each procedure differed from first recommendations (Table 4). GPs were significantly more likely to consider gland excision than were PGs. PGs more frequently recommended use of periosteal anchoring (either alone or in combination with pocketing) as compared to their initial treatment suggestions (Figure 1). Of those who initially chose pocketing, 45% proposed the same technique for revision.

A significant number of respondents suggested discussing the possibility of surgical failure with owners with no difference between groups (Table 5). However, PGs were significantly more likely to note the risk of prolapse in the contralateral eye and marginally more likely to state they would discuss possible keratoconjunctivitis sicca (KCS) sequelae in the operated eye.

Nearly a quarter (n = 47, 23.4%) of GPs offered referral for initial and/or revision replacement surgery; of those, 23 indicated that they did not have proficiency in surgical replacement. Six GPs who had recommended periosteal anchoring for initial or revision surgery specified referral for that procedure. Four of 20 individuals who indicated unavailable treatments indicated that they had no ability to refer to a veterinary ophthalmologist.

### 3.4. FHV-1 Keratitis

**Figure 2:**
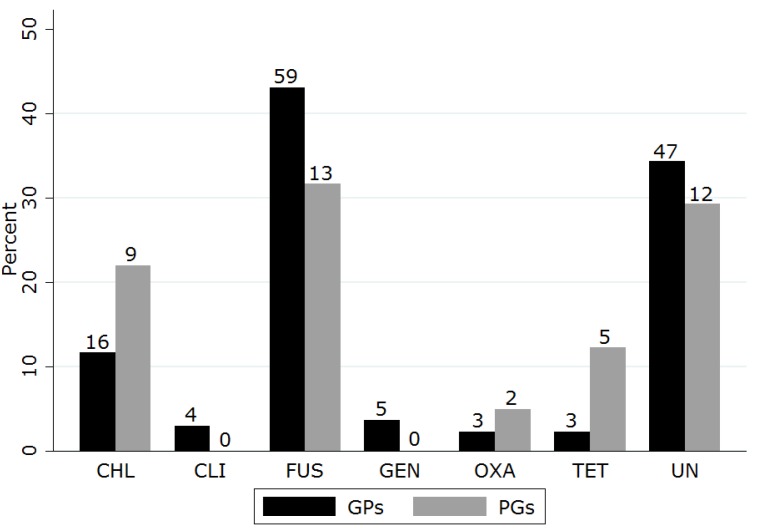
Topical antibiotic choices for FHV-1 keratitis selected by UK general practitioners and postgraduate groups who suggested topical antibiotic therapy in a questionnaire-based survey (CHL, chloramphenicol; CLI, clindamycin; FUS, fusidic acid; GEN, gentamycin; OXA, oxacillin; TET, tetracyclines; UN, unspecified; numbers indicate number of respondents suggesting treatment).

**Figure 3:**
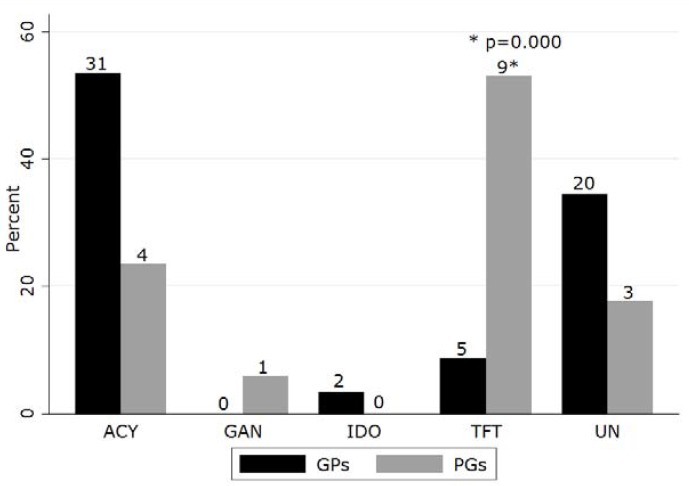
Topical antiviral choices for FHV-1 keratitis selected by UK general practitioners and postgraduate groups who suggested topical antiviral therapy in a questionnaire-based survey (ACY, acyclovir; GAN, ganciclovir; IDO, idoxuridine; TFT, trifluorothymidine; UN, unspecified; numbers indicate number of respondents suggesting treatment).

When presented with the case of feline dendritic keratitis, the majority of both groups recommended the use of a topical antibiotic (Table 6). Topical antiviral agents and topical lubricants were recommended by a third of respondents with no significant difference between groups. There were few recommendations for topical NSAIDs or autologous serum. A variety of topical antibiotics were nominated (Figure 2). Fusidic acid was most frequently suggested, followed by chloramphenicol and tetracyclines, with no significant differences found between groups for any agent. Similarly, a number of topical antiviral drugs were suggested (Figure 3). A significantly higher proportion of PGs recommended topical trifluorothymidine than did GPs (chi square *p* = 0.000).

Suggested systemic therapies for FHV-1 keratitis were more varied between the two groups. PGs were significantly more likely to nominate a systemic antiviral (famciclovir when specified) and lysine supplement (Table 7). Slightly more than a third of all respondents suggested a systemic nonsteroidal anti-inflammatory (NSAID), often citing analgesia, with a smaller number of respondents recommending interferon or systemic antibiotics (amoxicillin-clavulanate n = 7, doxycycline n = 12, clindamycin n = 1, unspecified n = 9).

**Figure 4:**
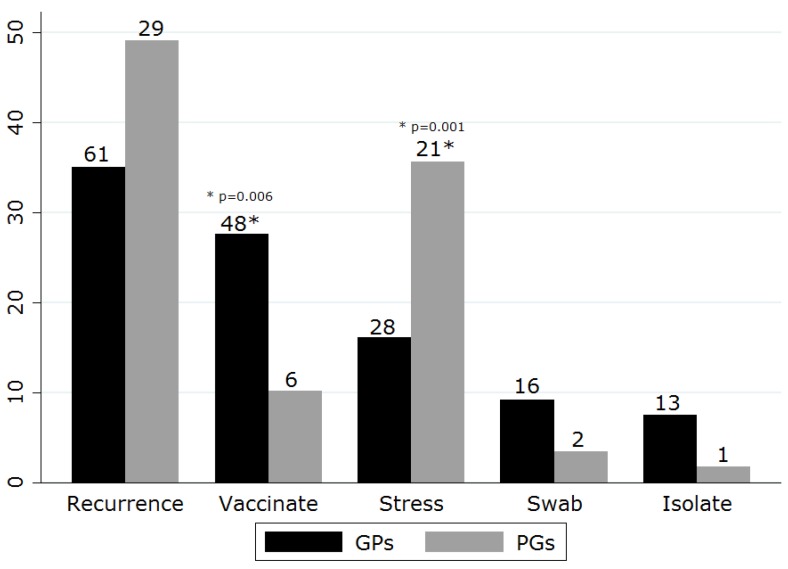
Additional recommendations for FHV-1 keratitis as nominated by general practitioners and postgraduate groups based in the UK in a questionnaire-based survey (Recurrence, discussed likelihood of recurrence; Vaccinate, discussed need for regular vaccination; Stress, discussed stress/environmental triggers; Swab diagnostics, recommended submission of ophthalmic swab for diagnostic testing; Isolate, discussed contagion to other cats and/or recommended isolation). Numbers indicate number of respondents making suggestion.

Additional recommendations for treatment were varied. More than 5% of respondents recommended discussing recurrence, environmental stress, and issues of contagion (Figure 4). GPs were more likely to recommend regular vaccination (chi square *p* = 0.006) whilst PGs were more likely to discuss stress avoidance (chi square *p* = 0.001). Few respondents recommended diagnostic testing for FHV-1; when specified, polymerase chain reaction (PCR) and/or virus isolation was frequently recommended. In addition to these top five recommendations, 32 additional suggestions were offered by <5% of respondents which covered surgery (debridement, keratotomy, third eyelid, and conjunctival flaps), atropine, FeLV/FIV testing, vaccination of contacts, homeopathy, contact lens, steroids, cyclosporine, deworming, vitamin E, lecithin, and recommendations to avoid vaccination. All recommendations aside from the top five were suggested only by the GP group with the single exception of one PG who suggested deworming. 

Recommendations for relapse did not differ substantially from initial treatment suggestions. The majority of respondents suggested repeating their initial treatment advice (71.3% GPs, 81.5% PGs). A small number of respondents who had not previously suggested surgery (n = 20), antiviral therapy (n = 15), lysine supplement (n = 7), interferon (n = 9), or systemic antibiotic therapy (n = 3) did so for relapsed cases. No new recommendations for topical antibiotics were made, although a few respondents (n = 3) recommended changing to a different antibiotic. Suggested surgical procedures were keratectomy, debridement, third eyelid flaps, conjunctival flaps, and enucleation.

A number of respondents indicated there were herpesvirus treatments which were unavailable to them (25.5% GPs, 16.1% PGs). More than half of these (55.5%) suggested that access to antiviral drugs was limited; although most respondents did not name specific agents, those cited were trifluorothymidine (n = 4), famciclovir (n = 3), cidofovir (n = 1), and idoxyuridine (n = 1).

## 4. Discussion

There appeared to be variation in treatment recommendations elicited by clinical vignettes of PNG and FHV-1 keratitis, which could potentially impact on the consistency of care given to animals affected by these conditions. Prior work has demonstrated treatment variation in cardiac, endocrine, and ocular diseases of companion animals [15,18,19,20]. This study provides additional evidence for such variation: although suggestions for initial treatment of PNG were generally consistent amongst all veterinarians, approaches to surgical failure varied more between GPs and PGs. Moreover, a wider range of treatments were suggested for FHV-1 keratitis, with larger discordance between GPs and PGs in the use of systemic agents.

Most respondents suggested surgical replacement of PNG, although more GPs trialed medical therapy prior to surgical intervention. Most chose a pocket procedure for initial repair (when the technique was specified). Although a variety of techniques for gland replacement have been published [7,21,22,23,24,25,26,27,28], there is limited data for comparative efficacy on surgical and lacrimal outcomes, particularly for breeds thought to be at higher risk for recurrence or for development of KCS. Morgan’s pocket technique is considered technically less challenging than some other procedures and is frequently covered in ophthalmology surgical texts [3,4,5,6,29], factors which may have driven popularity amongst respondents. Our finding that periosteal anchoring was suggested more frequently by PGs for revision surgery suggests that it may be favored in patients more prone to recurrence, a view reinforced by some authors [28,30] and a recent study showing decreased recurrence in English Bulldogs when pocketing was augmented with a periosteal tack [22]. It is noteworthy that some of the GPs in our survey suggested referral specifically for periosteal anchoring, suggesting less comfort with the surgical technique in that group. 

A significantly greater number of GPs considered gland excision in the case of first surgery failure. Gland excision, though commonly recommended in the past [31], is currently discouraged due to published evidence of concomitant reduction of tear production [32,33,34,35]. Although a retrospective study associated excision with subsequent development of KCS [7], a number of respondents who suggested excision as a treatment option stated that they had never encountered this complication. KCS risk varies by sex, breed, and age [36,37,38]. It is likely that excision-related KCS may similarly vary and that willingness to excise may reflect experience with patient mix that is not fully captured by the published literature. Alternatively, since prolonged prolapse may also be associated with higher KCS risk [7], respondents may have suggested excision to serve owner cosmetic and financial preferences, rather than lacrimal function. Finally, since onset of KCS often occurs years after excision (mean 3.06 years, median 4.5 years [7]), it is possible that clinicians who did not report this complication may have been biased by shorter follow-up times. 

Currently and at the time of this survey, there are no approved veterinary pharmaceutical products for the treatment of FHV-1 keratitis. Suggested therapeutics have generally been derived from in vitro efficacy studies, experimental infection, and case series reports [8,39]. Topical antibiotics (recommended by the majority of all respondents) are used in both human and feline keratitis primarily for the prevention and treatment of secondary infection [8,9,40,41]. Although the difference did not reach statistical significance, more PG respondents recommended use of a lubricant. We speculate that lubricants may have been suggested due to tear film abnormalities documented in cats experimentally infected with FHV-1 [42], as well as to improve ocular comfort [30].

Although a similar proportion of PGs and GPs recommended a topical antiviral, product choice was disparate between groups, with a larger proportion of PGs suggesting trifluorothymidine. This may be due, in part, to limited availability of some topical preparations in the UK; trifluorothymidine must be obtained through the single national ophthalmic compounding pharmacy in the UK. However, aside from a controlled trial of cidofovir [43], topical antiviral efficacy has generally been deduced from in vitro and uncontrolled observational data [39]; perhaps as a consequence, disparate product recommendations are common in veterinary references [5] (pp. 396–399) [30] (p. 250) [44] (p. 470).

General practitioners and PGs diverged more dramatically in their systemic FHV-1 therapy recommendations, notably in the greater popularity of famciclovir and lysine amongst PGs. At the time of this survey, preliminary experimental safety and efficacy data for famciclovir in feline FHV-1 had been published, along with a small case series [45,46,47]. However, famciclovir therapy had not yet been included in contemporary texts or was discouraged due to safety concerns [48] (p. 145) [30] (p. 72) [44] (p. 470). Lysine recommendations also varied between authors at the time of this survey. The European Cat Advisory Board included lysine as a recommended antiviral agent in their 2009 guidelines [9] whilst a contemporary evidence-based management guide suggested that lysine was futile at best and could potentially worsen disease and viral shedding [8]. Two recent systematic reviews summarizing evidence available at the time of this survey have also suggested no evidence for lysine in prevention or treatment of FHV-1 or prevention of human herpes simplex labialis [49,50]. However, lysine is still considered potentially beneficial by some veterinary ophthalmologists and virologists [39,51]. Our survey was not designed to elicit reasons for variation but we speculate that the lower number of lysine suggestions from GPs might reflect differences in information sources, evidence appraisal, or product availability between the two groups.

Although stress avoidance and recognition of FHV-1 chronicity emerged as consensus themes amongst respondents, vaccine recommendations varied by practitioner group. Current vaccine guidelines vary in suggested FHV-1 vaccine intervals due to non-sterilizing immunity and uncertainty regarding duration of immunity [9,52]; some suggest that FHV-1 may recrudesce in latent carriers following modified live FHV-1 vaccination [51,53]. Thus, practitioner recommendations may vary depending on information source. Finally, we were struck by numerous and varied additional recommendations for management of FHV-1 keratitis. It has been suggested that treatment proliferation occurs for chronic disease in which little is known and empirical therapy forms the basis for practice [54] (p. 63).

Treatment variation in human medicine is greater in areas with larger evidence gaps and for conditions which lack clinical guidelines [10]. Additionally, physician social networks have been shown to drive regional variation in prostate cancer and coronary artery disease care in the United States [55,56]. In veterinary medicine, few high quality clinical trials are available, constraining information sources to lower levels of evidence [57]. In this environment, information sources, social networks, and client preferences may drive care more substantially and further work in identifying the sources of veterinary treatment variation is needed. While guidelines may help reduce heterogeneity in clinical decision making, they are ideally formulated using best available evidence alongside inclusion of all stakeholders into the guideline process. Our results, combined with the accompanying survey of current evidence, suggest that there is need for both guidelines for companion animal ocular disease and additional research to establish optimal treatment for these conditions. In the low resource setting of veterinary medicine, electronic medical records could be leveraged to collect multicentre cohort data, create patient registries, and serve as the basis of pragmatic clinical trials.

## Figures and Tables

**Table 1:** Demographic and vignette question response rates.

Question	Overall n	Overall %	GP n	GP %	PG n	PG %
Survey	326		266		60	
Age	231	70.9	171	64.3	60	100
Gender	231	70.9	171	64.3	60	100
Graduation year	230	70.6	170	63.9	60	100
Initial prolapse treatment	261	80.0	201	75.6	60	100
Prolapse surgical failure treatment	184	56.4	129	48.5	55	91.7
Nictitans additional recommendations	182	55.8	128	48.1	54	90.0
FHV-1 initial treatment	233	71.5	174	65.4	59	98.3
FHV-1 relapse	214	65.6	160	60.2	54	90.0
FHV-1 additional recommendations	233	71.5	174	65.4	59	98.3

**Table 2:** Demographic characteristics of GP and PG respondents.

	Overall n	Overall	GP n	GP	PG n	PG	*p* Value
**Gender**							
Male	96	41.6%	66	38.6%	30	50.0%	0.123
Female	135	58.4%	105	61.4%	30	50.0%	
No answer given	95		95		0		
**Age**							
Median age (all)	230 *	40	171	39 years	59	40 years	0.2151
Median age (male)	94	44	65	45 years	29	42 years	0.5335
Median age (female)	135	36	105	36 years	30	39.5 years	0.0497
No answer age and/or gender	97		96		1		
**Year of qualification**							
Median year qualified (all)	230	1996.5	170	1997	60	1995	0.1227
Median year qualified (male)	95	1993	65	1992	30	1994.5	0.9553
Median year qualified (female)	134	1999	104	2000	30	1996.5	0.0541
No answer year and/or gender	97		97		0		

* One individual who answered age question provided a range rather than discrete number thus could not be included in summary data analysis.

**Table 3:** Canine PNG treatments recommended by GP and PG groups responding to a vignette questionnaire.

Treatments	Overall n	Overall %	GP n	GP %	PG n	PG %	*p* Value
**Total respondents**	261		201		60		
**All replacement surgery**	251	96.2	191	95.0	60	100	0.078
Pocket	125	47.9	75	37.3	50	83.3	* 0.000
Pocket or anchor	8	3.1	5	2.5	3	5.0	**^ǂ^** 0.390
Periosteal anchor	6	2.3	6	3.0	0	0	**^ǂ^** 0.342
Unspecified surgery	112	42.9	105	52.2	7	11.7	* 0.000
**Excision option**	24	9.2	24	11.9	0	0	* 0.005
**Excision only**	9	3.4	9	4.5	0	0	**^ǂ^** 0.124
**Medical trial**	59	22.6	52	25.9	7	11.7	0.021

* Significant with Bonferroni corrected *p* < 0.05; bolded *p* values are significant after correction for multiple comparison testing; **^ǂ^** Fisher’s exact test.

**Table 4:** Canine PNG surgical failure treatments recommended by GP and PG groups responding to a vignette questionnaire.

Treatments	Overall n	Overall %	GP n	GP %	PG n	PG %	*p* Value
**Total respondents**	184		129		55		
**All replacement surgery**	166	90.2	112	86.8	54	98.2	* 0.018
Pocket	51	27.7	31	24.0	20	36.4	0.087
Periosteal anchor	20	10.9	11	8.5	9	16.4	0.118
Pocket or anchor	4	2.2	0	0	4	7.3	*^,ǂ^ 0.007
Pocket +/− anchor	5	2.7	0	0	5	9.1	* 0.001
Pocket + anchor	7	3.8	2	1.6	5	9.1	0.014
All techniques with anchoring	36	19.6	13	10.1	23	41.8	* 0.000
Perilimbal pocket (Prémont)	1	0.5	0	0	1	1.8	^ǂ^ 0.299
Unspecified surgery	78	42.4	68	52.7	10	18.2	* 0.000
**Excision option**	37	20.1	36	27.9	1	1.8	* 0.000
**Excision only**	10	5.4	10	7.8	0	0	^ǂ^ 0.034

* Significant with Bonferroni corrected *p* < 0 05; bolded *p* values are significant after correction for multiple comparison testing. ^**ǂ**^ Fisher’s exact test.

**Table 5:** Additional PNG management recommendations by GP and PG groups responding to a vignette questionnaire.

Recommendation	Overall n	Overall %	GP n	GP %	PG n	PG %	*p* Value
Total respondents	182		128		54		
Discuss risk of prolapse in contralateral eye	69	37.9	40	31.3	29	53.7	* 0.004
Warn owner of surgical failure	58	31.9	44	34.4	14	25.9	0.264
Discuss monitoring for KCS	50	27.5	29	22.7	21	38.9	0.025
Recommend prophylactic surgery contralateral eye	7	3.8	3	2.3	4	7.4	^ǂ^ 0.198

* Significant with Bonferroni corrected *p* < 0.05. **^ǂ^** Fisher’s exact test.

**Table 6:** FHV-1 keratitis topical treatments recommended by GP and PG groups responding to a vignette questionnaire.

Treatments	Overall n	Overall %	GP n	GP %	PG n	PG %	*p* Value
Total respondents	233		174		59		
Antibiotic	178	76.4	137	78.7	41	69.5	0.148
Antiviral	75	32.2	58	33.3	17	28.8	0.521
Lubricant	77	33.0	52	29.9	25	42.4	0.078
NSAID ^**1**^	8	3.4	8	4.6	0	0.0	^ǂ^ 0.207
Autologous serum	9	3.9	8	4.6	1	1.7	^ǂ^ 0.455

^ǂ^ Fisher’s exact test. ^1^ nonsteroidal anti-inflammatory.

**Table 7:** FHV-1 keratitis systemic therapies recommended by GP and PG groups responding to a vignette questionnaire.

Treatments	Overall n	Overall %	GP n	GP %	PG n	PG %	*p* Value
**Total respondents**	233		174		59		
**NSAID**	80	34.3	59	33.9	21	35.6	0.814
Meloxicam	41	17.6	34	19.5	7	11.9	
Carprofen	1	0.4	1	0.6	0	0	
Unspecified	38	16.3	24	13.8	14	23.7	
**Lysine**	75	32.2	46	26.4	29	49.2	* 0.001
**Antiviral**	68	29.2	30	17.2	38	64.4	* 0.000
Famciclovir	65	27.9	27	15.5	38	64.4	
Unspecified	3	1.3	3	1.7	0	0	
**Interferon**	33	14.2	24	13.8	9	15.2	0.781
**Antibiotic**	29	12.4	23	13.2	6	10.2	0.540

* Significant with Bonferroni corrected *p* < 0.05.

## Supplementary Materials

The following are available online at http://www.mdpi.com/2306-7381/5/2/54/s1, Questionnaire 1: Online questionnaire, Questionnaire 2: British Association of Veterinary Ophthalmologists Winter Meeting questionnaire.
