# Supplementary material for: Variation in the Reported Management of Canine Prolapsed Nictitans Gland and Feline Herpetic Keratitis
Source: Vet Sci. 2018 Jun 1;5(2):54. doi: 10.3390/vetsci5020054 (PMC6024657; doi:10.3390/vetsci5020054)
Supplement: Supplementary file 1 [file vetsci-05-00054-s001.zip › Online questionnaire.pdf]

Questionnaire 1. Online Survey Monkey questionnaire

# The use of diagnostic tools and treatment options for ophthalmology

## Welcome to the Survey!

Thank you for taking the time to participate in this survey.
It should take no longer than 10 minutes. Your answers are greatly appreciated.

***1. Do you currently undertake any small animal work?**

- Yes
- No

# The use of diagnostic tools and treatment options for ophthalmology

## Diagnostic Tools

**2. What type of diagnostic tools are available at your practice for investigating ophthalmology cases?**
**Which of these tools do you use on a regular basis?**

**Please tick all that apply.**

| | Have in my practice | Use regularly (at least once a month) |
|---|---|---|
| Penlight | ☐ | ☐ |
| Condensing lens | ☐ | ☐ |
| Magnifying loupe or otoscope with speculum removed | ☐ | ☐ |
| Direct ophthalmoscope | ☐ | ☐ |
| Schirmer Tear Test | ☐ | ☐ |
| Ophthalmic stains such as Fluorescein and Rose Bengal | ☐ | ☐ |
| Schiotz tonometer | ☐ | ☐ |
| Tonometer e.g. TonoPen | ☐ | ☐ |
| Slit lamp | ☐ | ☐ |
| Indirect ophthalmoscope | ☐ | ☐ |
| Gonioscopy lens | ☐ | ☐ |
| Finoff ocular transilluminator | ☐ | ☐ |

# The use of diagnostic tools and treatment options for ophthalmology

## Case 1

Please read the following case information and then answer the questions below.

Owner: Mr Davids
Animal: Lola
Species: Canine
Breed: Lhasa Apso
Sex: Female
Age: 9 months
Weight: 5 kg
Insurance status: Insured

History: Mr Davids noticed yesterday that Lola has an abnormal pink mass by the medial canthus of her left eye. He says Lola has never had this problem before. Lola is bright and lively within herself.

Clinical Examination: Upon examination, you identify that the pink mass is the tear gland. There are no other lesions on the eye and the result of a Schirmer tear test is normal bilaterally (>18mm/min OU). Forceps examination rules out any deformity of the cartilage of the third eyelid and confirms the diagnosis of nictitans gland prolapse (cherry eye). Your full clinical exam proves Lola to be otherwise healthy.

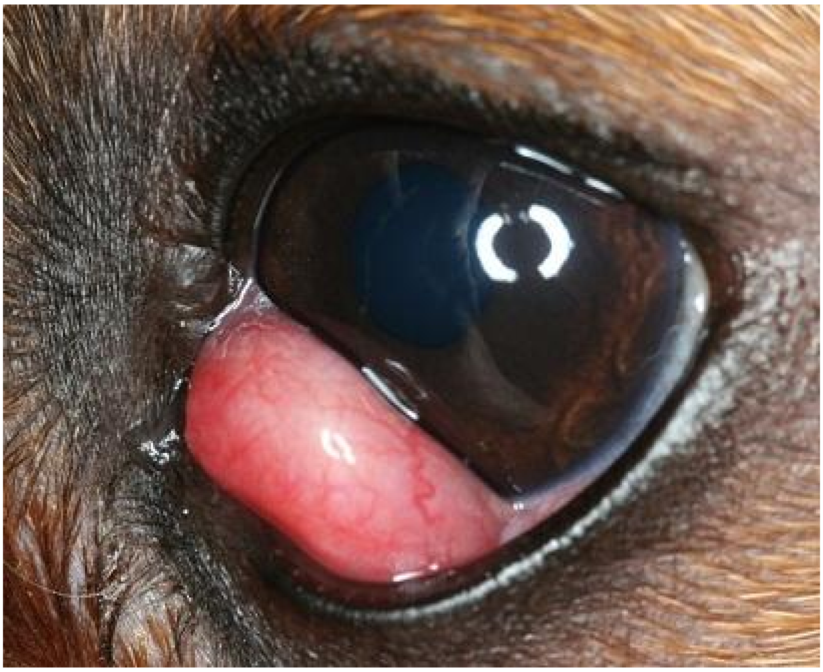

### 3. What would be your initial treatment choice? Please include details of any drugs or techniques you may use.

| | |
|---|---|
| Treatment 1 | |
| Treatment 2 | |
| Treatment 3 | |
| Treatment 4 | |

**4. Would you make any other recommendations to the owner regarding the longer term management of this case?**

- Yes
- No

**5. If yes, please state what you would recommend:**

**6. How would you treat the condition should it relapse?**

**7. Are there any treatments you would like to give but are not available to you at your practice?**

- No - all treatments I'd use are available at my practice
- Yes - there are treatments I'd like to use but are unavailable to me at my practice

**8. If yes, please state which treatments you would like to give that are unavailable to you currently:**

# The use of diagnostic tools and treatment options for ophthalmology

## Case 2

Please read the following case information and then answer the questions below.

Owner: Miss Crosby
Animal: Scoobie
Species: Feline
Sex: Male
Breed: DSH
Age: 3 years
Weight: 4 kg
Insurance status: Insured.

History: Miss Crosby adopted Scoobie as a kitten from a rescue shelter. As a kitten he had 'flu-like' symptoms which have recurred occasionally. Miss Crosby had him vaccinated when she adopted him but has not kept up with annual boosters. Over the last 7 days, his right eye has become closed and painful with a watery discharge.

Clinical examination: Upon examination, Scoobie's lesions are confined to just the right eye. Superficial branching corneal neovascularisation is visible with the presence of several small separate geo-dendritic ulcers which become obvious using Fluorescein stain.
There is no evidence of entropion and your Schirmer tear test has proved normal (15mm/min). Due to the presence of the dendritic ulcer you diagnose feline herpes virus (FHV-1).

**9. What would be your initial choice of treatment? Please include details of any drugs or techniques you may use.**

Treatment 1

Treatment 2

Treatment 3

Treatment 4

**10. Would you make any other recommendations regarding the longer term management of this case?**

- Yes
- No

**11. if yes, please state what you would recommend:**

**12. How would you treat the condition should it relapse?**

**13. Are there any treatments you would like to give but are not available to you at your practice?**

- No - all treatments I'd use are available at my practice
- Yes - there are treatments I'd like to use but are unavailable to me at my practice

# The use of diagnostic tools and treatment options for ophthalmology

**14. If yes, please state which treatments you would like to give that are unavailable to you currently:**

## Sources of Information

**15. What sources of information would you use for advice and guidance when diagnosing and managing ophthalmology cases?**

**Please rank your preferences, placing 1 beside the most important source, 2 beside the next most important source, continuing on through to 10 beside the least most important source.**

| | |
|---|---|
| Personal notes or files | |
| Consult a specialist by telephone | |
| Promotional literature | |
| Other first opinion veterinary surgeons | |
| Articles in journals | |
| CPD courses | |
| Veterinary internet sites | |
| Attending conferences | |
| Textbooks | |
| Representatives from companies | |

**16. Please list 3 factors that contribute most to your decision making about the diagnosis and treatment of a clinical ophthalmology case.**

1.

2.

3.

# The use of diagnostic tools and treatment options for ophthalmology

## Information about you

**17. What is your gender?**

- ○ Male
- ○ Female

**18. What is your age in years?**

**19. In what year did you graduate?**

**20. Do you have, or are you working towards a certificate in veterinary ophthalmology?**

- ○ Yes
- ○ No

**21. If you would like to be entered into the prize draw for a £80 voucher of your choice, please enter your email address in the box below. Your details will be removed from the questionnaire so that your answers cannot be identified as your own**

**22. Would you be interested in receiving a summary of the results from this project?**

- ○ Yes
- ○ No

**23. Please enter your email address below, if you haven't already written it in the previous box. We will email you a short summary of our results upon completion of the project.**

**24. If you have any comments about the cases or questionnaire in general, please comment below.**

## Thank You!

This is the end of the questionnaire.

Thank you for taking the time to fill out the questionnaire. If you have any questions please do not hesitate to get in touch either with myself or my supervisor, Marnie Brennan. We are contactable via:

Sarah Baker: svydsjb1@nottingham.ac.uk
Marnie Brennan: marnie.brennan@nottingham.ac.uk
